# Investigating spatial specificity and data averaging in MEG

**DOI:** 10.1016/j.neuroimage.2009.07.043

**Published:** 2010-01-01

**Authors:** Matthew J. Brookes, Johanna M. Zumer, Claire M. Stevenson, Joanne R. Hale, Gareth R. Barnes, Jiri Vrba, Peter G. Morris

**Affiliations:** a Sir Peter Mansfield Magnetic Resonance Centre, School of Physics and Astronomy, University of Nottingham, University Park, Nottingham NG7 2RD, UK; b Wellcome Trust Centre for Neuroimaging, University College London, London, UK; c Elekta-Neuromag Oy, Helsinki, Finland

**Keywords:** MEG, Spatial specificity, Spatial resolution, Source localisation, Linearly constrained minimum variance beamformer, Retinotopic mapping, Regularisation, Information content

## Abstract

This study shows that the spatial specificity of MEG beamformer estimates of electrical activity can be affected significantly by the way in which covariance estimates are calculated. We define spatial specificity as the ability to extract independent timecourse estimates of electrical brain activity from two separate brain locations in close proximity. Previous analytical and simulated results have shown that beamformer estimates are affected by narrowing the time frequency window in which covariance estimates are made. Here we build on this by both experimental validation of previous results, and investigating the effect of data averaging prior to covariance estimation. In appropriate circumstances, we show that averaging has a marked effect on spatial specificity. However the averaging process results in ill-conditioned covariance matrices, thus necessitating a suitable matrix regularisation strategy, an example of which is described. We apply our findings to an MEG retinotopic mapping paradigm. A moving visual stimulus is used to elicit brain activation at different retinotopic locations in the visual cortex. This gives the impression of a moving electrical dipolar source in the brain. We show that if appropriate beamformer optimisation is applied, the moving source can be tracked in the cortex. In addition to spatial reconstruction of the moving source, we show that timecourse estimates can be extracted from neighbouring locations of interest in the visual cortex. If appropriate methodology is employed, the sequential activation of separate retinotopic locations can be observed. The retinotopic paradigm represents an ideal platform to test the spatial specificity of source localisation strategies. We suggest that future comparisons of MEG source localisation techniques (e.g. beamformer, minimum norm, Bayesian) could be made using this retinotopic mapping paradigm.

## Introduction

Magnetoencephalography (MEG) is a non-invasive functional brain imaging modality that measures the magnetic fields induced above the scalp by the flow of ion currents between the dendritic tree and the soma of neuronal cells (Hamalainen et al., 1993). MEG is a direct measure of neuronal activity since the magnitude of the measured fields is directly proportional to the magnitude of the neural current. This means that MEG has excellent temporal resolution. However, its spatial specificity (*defined here as the ability to extract accurate temporal estimates of electrical activity from spatially separate sources in the brain*) is limited due to the inverse problem which can be stated: *given a measured magnetic field distribution outside the head, can we reconstruct spatially the neuronal current distribution in the brain*? This problem is ill-posed (Hadamard, 1952) since, due to field cancellation, a single measured field could result from an infinite number of possible current distributions, meaning that no unique inverse solution exists. However, in recent years the spatial specificity of MEG has been improved by the introduction of beamformer techniques (Van Drongelen et al., 1996; Van Veen et al., 1997; Robinson and Vrba, 1998; Gross et al., 2001; Sekihara et al., 2001, 2002b).

Beamforming is a spatial filtering approach based on a weighted sum of field measurements (Van Drongelen et al., 1996; Van Veen et al., 1997; Robinson and Vrba, 1998; Gross et al., 2001, Sekihara et al., 2001, 2002b; Robinson, 2004; Cheyne et al., 2006). Each measured field is multiplied by a weighting factor, and the weighted sum of sensor measurements gives an estimate of source amplitude at a predetermined location in the brain. Unlike other localisation metrics, weighting parameters are data driven and derived based on power minimisation: the average power emanating from the source space (i.e. the head) within a given time frequency window (the *covariance window*) is minimised, but with a constraint that power originating from our predetermined location must remain in the output signal. The result is a set of weighting parameters tuned specifically to a single location. Sequential application of this calculation to a number of locations can result in a volumetric image of source power. Task related change in activity can be imaged either by contrasting power estimates made using separate active and control covariance windows, or by parsing the beamformer projected time series into active and control segments using beamformer coefficients derived from a single covariance window. When processed in this way the spatial specificity of MEG is dependant on the spatial specificity of the weighting parameters. This in turn depends on the signal to noise ratio (SNR) of the data, the duration of the covariance window (*T*_cov_), the signal bandwidth (BW), and matrix regularisation (Brookes et al., 2008). Some of these factors can be controlled by experimental design, i.e. *T*_cov_. Other factors, i.e. BW, SNR etc. depend on the effect of interest.

Neuromagnetic effects fall generally into two groups (Pfurtscheller and Lopes da Silva, 1999). *Non-phase-locked induced effects* comprise stimulus related changes in spontaneous rhythms. Due to their non-phase-locked nature they are eliminated by trial averaging. *Phase-locked effects* comprise evoked responses and driven oscillations. In both cases the onset phase of the neuromagnetic signal is equivalent for each stimulus presentation meaning that the effect remains visible after averaging. Beamformers are well suited to imaging induced responses. This is because induced power changes can last for several seconds and, in some cases, their duration can be controlled by experimental design. The covariance window is constructed by concatenating data from multiple trials and if the effect of interest remains observable and stationary for an extended period within each trial, the concatenated covariance window is large. Beamformers are sensitive to covariance window duration and the extended period helps to achieve an accurate spatial reconstruction. Averaging phase-locked effects across trials allows a larger SNR than that of induced effects, which potentially could improve spatial specificity of beamformer weights. However, averaging (rather than concatenating) data from individual trials reduces the covariance window. In addition, the reduction in random noise achieved by averaging can cause covariance matrices to become singular or ill-conditioned meaning that matrix regularisation has to be introduced. These effects make imaging averaged phase-locked responses difficult. SAMerf (Robinson, 2004) and erSAM (Cheyne et al., 2006, 2007) are beamformer techniques that have been used to image evoked responses. In both cases, weighting parameters are derived based on unaveraged data. This is sensible since *T*_cov_ is made large, however, one could argue that the weights are not tuned optimally to the effect of interest and spatial specificity is not optimal.

In this paper we aim to combine known theory, simulation and experiment in order to investigate the improved spatial specificity that may be afforded by the increase in SNR observed when sensor level MEG data are averaged. Initially, a simple model is considered comprising a dipolar source and Gaussian noise. Previously published theory (Brookes et al., 2008) is used to show that the accuracy of a data derived covariance matrix is affected by trial averaging. The effect of averaging on matrix condition number and techniques for matrix regularisation are also considered. This simulation is extended to a 2-source simulation employing measured MEG noise. We show that in most cases, averaging across trials will not significantly change results. However, if two phase-locked sources are in close proximity, averaging across trials can improve spatial specificity.

Our simulated results are extended to a retinotopic mapping experiment. A smoothly rotating and flashing wedge visual stimulus is employed to induce driven, phase-locked oscillations within visual cortex. It is well known that the visual field is mapped retinotopically onto the visual cortex, meaning that as the wedge moves, cortical pyramidal cells at different locations are activated sequentially. This gives the impression of a moving electrical source in the brain. Tracking the retinotopic motion of such a source has been done previously (Engel et al., 1994; Aine et al., 1996; Gramfort et al., 2007; Yoshioka et al., 2008; Hagler et al., 2009); however it remains challenging in MEG due to the non-stationary nature of the source, and the complex geometry of the visual cortex. We present results showing that this moving source, induced by the retinotopic stimulus, can be tracked using MEG beamformer methodology. A sliding covariance window technique, similar to that introduced by Dalal et al. (2008), is used to extract timecourse estimates showing the sequential activation of regions in visual cortex. We show that these results are only possible if data averaging is employed. The excellent spatiotemporal resolution of MEG is highlighted, and we propose that the retinotopy paradigm be used as a future measure of spatial specificity.

## Theory and simulation

### Notation

Using a beamformer, electrical source amplitude, *Q̂_**θ**_*(*t*) at a predetermined location in source space is estimated using a weighted sum of the field measurements such that:

(1) $${\hat{Q}}_{\theta }\left(t\right)=W_{\theta }^{T}m\left(t\right)$$

***m***(*t*) is a vector of magnetic field measurements made at *M* MEG sensors at time *t*. ***W***_***θ***_ is a vector of weighting parameters that are tuned to the location and orientation represented by ***θ***. Using a spherical head model, since MEG is insensitive to radial dipoles, source orientation can be represented by a vector in a plane tangential to the radial direction so that ***θ*** = [***r***,*δ*], where ***r*** represents location, and *δ* is the angle of the source with respect to the azimuthal direction. Note that using a multi sphere model (Huang et al., 1999), the tangential plane is derived with respect to the average sphere. To derive ***W***_***θ***_, the overall power in the estimated signal, $ɛ\left[{\hat{Q}}_{\theta }^{2}\right]$, computed over covariance window, *T*_cov_, is minimised but with the constraint that power originating from a source with location and orientation (***θ***) must remain in the output signal. Mathematically this can be written.

(2) $$\min_{W_{\theta }}\left[ɛ({\hat{Q}}_{\theta }^{2})\right]\text{ subject to }W_{\theta }^{T}L_{\theta }=1$$

where ***L***_***θ***_ represents the lead fields. The estimated power at ***θ*** can be represented by $ɛ\left[{\hat{Q}}_{\theta }^{2}\right]=W_{\theta }^{T}CW_{\theta }$ and **C** represents the *M* × *M* data covariance matrix. The solution to Eq. (2) is (Van Veen et al., 1997):

(3) $$W_{\theta }^{T}={\left[L_{\theta }^{T}C^{-1}L_{\theta }\right]}^{-1}L_{\theta }^{T}C^{-1}\text{.}$$

Eq. (3) is well known and shows clearly that the beamformer weighting parameters are dependent on an accurate measurement of data covariance. For all computations in this paper the scalar beamformer is employed. In this formulation the angle *δ* is found independently at each *r* using a non-linear search to compute the orientation of maximal variance (Robinson and Vrba, 1998).

### Analytical insights

Our previous work (Brookes et al., 2008) has investigated what parameters affect the accuracy of the covariance matrix. The covariance matrix error, Δ**C**, is defined as the difference between the measured covariance matrix, **C**, and a perfectly calculated covariance matrix, denoted by **C**_0_. Generally we can write:

(4) $$\Delta C=C-C_{0}\text{.}$$

Using a model incorporating a single dipolar source (amplitude *Q*_1_ and lead field pattern ***B***_1_) and Gaussian noise uncorrelated across sensors (variance *ν*^2^) the perfectly constructed covariance matrix is given by

(5) $$C_{0}=v^{2}I+Q_{1}^{2}B_{1}B_{1}^{T}$$

and the Frobenius norm of the covariance matrix error can be shown to be:

(6) $${\left\|\Delta C\right\|}_{F}=v^{2}M\sqrt{\frac{2\mathrm{SNR}+1}{2T_{\mathrm{cov}}BW}}\text{.}$$

*SNR* represents the signal to noise ratio of the source of interest and is given by:

(7) $$\mathrm{SNR}={Q_{1}^{2}\|B_{1}\|}_{F}^{2}/Mv^{2}\text{.}$$

The quantity ${\left\|\Delta C\right\|}_{F}$ is a measure of the total error in the covariance matrix, and previous simulations have shown that if this quantity is large, the accuracy of the beamformer estimated source power will be degraded severely (Brookes et al., 2008).

It is possible to use Eq. (6) to provide insight into the effect of data averaging on the accuracy of the covariance matrix. We know that by increasing the number of averages the random noise variance, *ν*^2^, will be reduced such that *ν*^2^ → *ν*^2^ / *N*_ave_ where *N*_ave_ is the total number of trials averaged. In addition, the covariance window duration will also be reduced such that *T*_cov_ → *T*_cov_ / *N*_ave_. By considering again Eq. (6) it is simple to show that the Frobenius norm of the covariance matrix error becomes:

(8) $${\left\|\Delta C\right\|}_{F}=v^{2}M\sqrt{\frac{2N_{\text{ave}}\mathrm{SNR}+1}{2N_{\text{ave}}T_{\mathrm{cov}}BW}}\text{.}$$

**Fig. 1:**
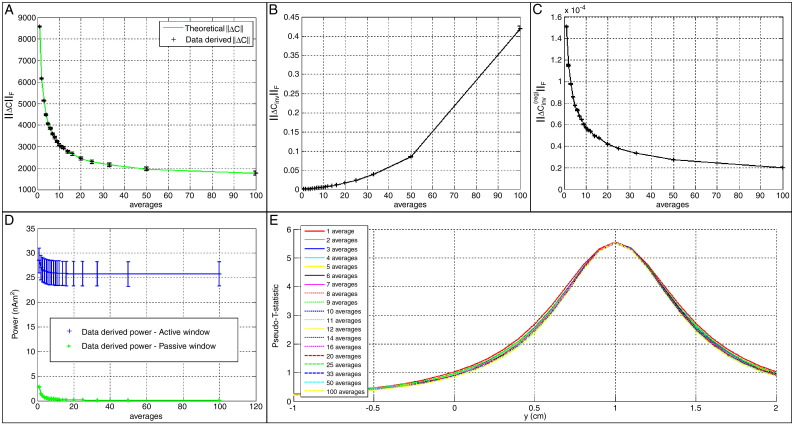
Results of the single source simulation. (A) ${\left\|\Delta C\right\|}_{F}$ versus *N*_ave_. The solid line shows the theoretical relationship (Eq. (6)) and the data points show values from the simulation. (B) ${\left\|\Delta C_{\text{inv}}\right\|}_{F}$ versus *N*_ave_ for the unregularised covariance matrix. (C) ${\left\|\Delta C_{\text{inv}}\right\|}_{F}$ versus *N*_*ave*_ for the regularised covariance matrix. Note the × 10^− 4^ scaling on the *y*-axis. (D) Beamformer projected power for the ON (blue) and OFF (green) periods. E) One dimensional beamformer pseudo-T-stat images extracted from a single interaction of the simulation. Images were formed in this case by scanning in the *y* direction but equivalent results were derived for both the *x* and *z* directions.

Plotting ${\left\|\Delta C\right\|}_{F}$ as a function of *N*_*ave*_ (see Fig. 1A, solid green line) shows that averaging across trials causes a slight reduction in ${\left\|\Delta C\right\|}_{F}$ as the number of averages is increased.

### Single source simulation

As evidenced by Eq. (3), the accuracy of beamformer weights is dependent not on the covariance matrix, but the inverse of the covariance matrix. This means that not only must the covariance estimate be accurate (i.e. ${\left\|\Delta C\right\|}_{F}$ should be small) but also that the inverse covariance matrix be accurate. With this in mind it becomes useful to introduce the inverse covariance matrix error, Δ**C**_inv_.

(9) $$\Delta C_{\text{inv}}=C^{-1}-C_{0}^{-1}$$

where **C**_0_^− 1^ is the exact covariance matrix inverse assuming an infinite number of samples, and **C**^− 1^ is the data derived covariance matrix inverse. Using the matrix inversion lemma and assuming an infinite amount of data, we can show that

(10) $$C_{0}^{-1}=\frac{1}{v^{2}}\left\{I-\frac{Q_{1}^{2}B_{1}B_{1}^{T}}{v^{2}+Q_{1}^{2}{\left\|B_{1}\right\|}_{F}^{2}}\right\}\text{.}$$

In this mathematical formulation, it is possible to measure the accuracy of the inverse covariance matrix by computing the Frobenius norm of the inverse covariance matrix error, ${\left\|\Delta C_{\text{inv}}\right\|}_{F}$.

The effect of data averaging on ${\left\|\Delta C\right\|}_{F}\text{ and }{\left\|\Delta C_{\text{inv}}\right\|}_{F}$ was investigated in simulation. All simulations were based on the third order gradiometer configuration of a 275 channel CTF MEG system (Vrba et al., 1991; Vrba and Robinson, 2001, 2002). The location of the head with respect to the sensor array was based on an experimental recording session and a multi sphere head model (Huang et al., 1999) was used. A single dipolar source was simulated in the occipital cortex, and oriented perpendicular to the radial direction, *δ* = 25°. The forward solution, ***B***_*Q*__1_, was based on the derivation by Sarvas (1987). A single simulated trial comprised 0.5 s of activity (ON) followed by 0.5 s rest (OFF). The source was given as a root mean square (r.m.s.) source strength of *Q*_1_ = 5 nAm and the source timecourse during the ON period was represented by a series of Gaussian random numbers. A total of 100 trials were simulated, and the timecourse for each trial was identical to imitate a phase-locked evoked signal. Gaussian noise data were added at the sensor level with r.m.s. amplitude *v* = 86.6 fT. The sampling frequency was set to 600 Hz, and the flat frequency spectrum of both the signal and noise meant that the total bandwidth was at the Nyquist limit.

The effect of data averaging was assessed by grouping and averaging trials. For completely averaged data, 100 groups, each comprising a single trial were taken and averaged. Alternative examples include, 50 groups each comprising 2 trials, or 25 groups each comprising 4 trials. In some cases the total number of trials used did not equal 100, for example 33 groups of 3 trials was used. However, in all cases the total number of trials was as close to 100 as possible. For completely unaveraged data, a single group comprising 100 trials was used. In all cases the number of averages used, *N*_ave_, was equal to the total number of groups, and this took values of 1, 2, 3, 4, 5, 6, 7, 8, 9, 10, 11, 12, 14, 16, 20, 25, 33, 50 and 100.

For each value of *N*_ave_, four covariance matrices were constructed: **C**_act_ was created using only data recorded during the ON period. **C**_pass_ was created using data recorded during the OFF period. $C_{m}=\frac{\left(C_{\text{act}}+C_{\text{pass}}\right)}{2}$ and represents the mean covariance of the ON and OFF periods. A regularised version of **C**_*m*_ was also constructed, denoted by **C**_*m*_^(reg)^. The strategy for regularisation is described in Appendix I and is summarised by the equation:

(11) $$C_{m}^{\left(\text{reg}\right)}=C_{m}+(\left(\mu +1)s^{\text{unav}}-s_{C_{m}}\right)I$$

where $s_{C_{m}}$ is the minimum singular value of **C**_*m*_ and *s*^unav^ is the minimum singular value of **C**^(unav)^ which is a covariance matrix constructed using the entire 100 trial unaveraged dataset (including the ON and OFF periods). Here, *μ* represents a parameter used to regularise the unaveraged covariance matrix, **C**^(unav)^.

Regularisation is known to reduce spatial specificity and so for the purposes of all computations in this paper, *μ* was set to zero. Using Eq. (5), a theoretical version of **C**_*m*_ was constructed as $C_{m0}=v^{2}I+\left(\frac{Q_{1}^{2}}{2}\right)B_{Q1}B_{Q1}^{T}$ where the $Q_{1}^{2}/2$ term accounts for the fact that this equation represents mean covariance in the ON and OFF periods. The inverse of **C**_*m*__0_ was also derived using Eq. 10.

The differences between the analytical and data derived covariance matrices were assessed. The Frobenius norm of the covariance matrix error and inverse covariance matrix error were plotted as a function of the number of averages. Beamformer weights were constructed using the regularised covariance $\left(W_{\theta }^{T}={\left[L_{\theta }^{T}C_{m}^{{\left(\text{reg}\right)}^{-1}}L_{\theta }\right]}^{-1}L_{\theta }^{T}C_{m}^{{\left(\text{reg}\right)}^{-1}}\right)$ and used to plot reconstructed power, calculated at the known source location, for the ON (*P*_ON_ = ***W******_θ_**^T^***C**_act_***W***_***θ***_) and OFF (*P*_OFF_ = ***W******_θ_**^T^***C**_pass_***W***_***θ***_) periods as a function of *N*_ave_. Finally, 1-dimensional images were created showing the spatial distribution of the pseudo-T-statistic across the source. The pseudo-T-statistic, *T*, is given by (Vrba and Robinson, 2001):(12)
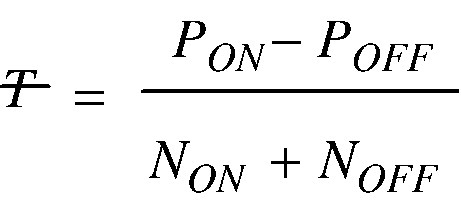
where *N*_ON_ and *N*_OFF_ represent the beamformer projected noise estimates in the ON and OFF periods respectively. The whole simulation was repeated 10 times and standard deviation between repeats plotted where appropriate. Note that in all cases, the number of data samples used to create covariance matrices was greater than the number of MEG channels. Note also that in all cases, the accuracy of the non-linear search for *δ* was assessed.

The results of the simulation are shown in Fig. 1. Fig. 1A shows ${\left\|\Delta C\right\|}_{F}$ as a function of *N*_ave_. The solid line shows the theoretical relationship (Eq. (6)) and the data points show the simulated values. The graph shows a modest improvement in the accuracy of the covariance matrix with averaging; furthermore, the theoretical relation agrees well with simulated data. Fig. 1B shows ${\left\|\Delta C_{\text{inv}}\right\|}_{F}$ as a function of *N*_ave_ for the unregularised case. As shown, without matrix regularisation, the accuracy of **C**_*m*_^− 1^ declines with data averaging. This is a result of a decrease in rank of the covariance matrix. As the number of averages is increased, the noise level decreases, this causes a corresponding drop in the smallest singular value of **C**_*m*_, which, in turn, causes an increase in condition number. As **C**_*m*_ becomes ill-conditioned, the accuracy of **C**_*m*_^−1 ^ is degraded. Fig. 1C shows ${\left\|\Delta C_{\text{inv}}\right\|}_{F}$ versus *N*_ave_ for the regularised case. Here the condition number of **C**_*m*_^(reg)^ is maintained approximately constant (see Appendix I) by the regularisation strategy outlined by Eq. (11). The result is that the accuracy of the inverse regularised covariance matrix is no longer degraded by data averaging. However, adding to the leading diagonal of the covariance matrix is equivalent to adding Gaussian noise to the simulation. This means that noise eliminated by data averaging has effectively been added back into the covariance matrix by matrix regularisation. Fig. 1D shows the beamformer projected power for the ON (blue) and OFF (green) periods as a function of the number of averages. Notice here that in all cases the active and control power estimates are approximately correct and are relatively insensitive to data averaging. This result is echoed in Fig. 1E which shows 1-dimensional pseudo-T-statistical images. (The simulated source is located at y = 1 cm) Like projected power, the beamformer image shows no dependency on whether or not the simulated data were averaged across trials. This lack of improvement is a result of regularisation and the fact that noise reduction through averaging is countered by the regularisation procedure. Unfortunately, as evidenced by Fig. 1B, regularisation is necessary to maintain the condition number. The overall conclusion is that in the limits of the single source and Gaussian noise model, averaging affords no benefit to beamformer reconstruction.

### Two source simulations

The above simulation was extended to incorporate a second source. Sources were located 1 cm apart in the occipital cortex, with parallel orientations in the tangential plane *δ* = 25°. A multi sphere head model was used and the location of the head with respect to the sensor array was based on an experimental recording session. The forward solutions were based on the derivation by Sarvas. For source 1, a single simulated trial comprised 0.5 s of activity followed by 0.5 s rest. For source 2, a single trial comprised 0.5 s of rest followed by 0.5 s of activity. This gave the impression that the two sources were activated sequentially. Source 1 was given r.m.s. amplitude 5 nAm. The r.m.s. amplitude of source 2 was set to either 5 nAm or 10 nAm. As for our single source simulation, activity comprised a timecourse of Gaussian random numbers. On each iteration of the simulation, 100 trials were simulated, and source timecourses were phase-locked. The simulation was repeated 10 times.

Two strategies for adding sensor noise were employed:

1. Gaussian noise data were added, equivalent to the single source simulation, with r.m.s. amplitude *υ* = 86.6 fT.
2. Experimental noise data were recorded from a single healthy volunteer.

To record experimental noise, a subject was asked to lie in the MEG system with their eyes open and 1000 s of resting state MEG data was recorded using the third order gradiometer configuration of a 275 channel CTF MEG system with a sampling rate of 600 Hz. (Note that 1000 s were recorded to allow 10 separate noise measurements, each 100 s in duration.) During data acquisition the location of the subject's head within the scanner was measured by energising 3 electromagnetic coils placed at three fiducial points on the head (nasion, left preauricular and right preauricular). Following data acquisition, the positions of these three coils were measured relative to the subject's head shape using a 3D digitiser (*Polhemus isotrack*). An MPRAGE structural MR brain image was acquired using a Philips Achieva 7 T MR system. (1 mm^3^ isotropic resolution, 256 × 256 × 160 matrix size, TR = 8.1 ms, TE = 3.7 ms, TI = 960 ms, shot interval = 3s, FA = 8° and SENSE factor = 2.) The locations of the three fiducial points, and the MEG sensors with respect to the brain anatomy was then determined by surface matching the digitised head surface to the head surface extracted from the MR image. These measured ‘brain noise’ data were then added to the simulated measurements from the two dipoles in the visual cortex.

As previously, data were grouped so that the effect of trial averaging could be assessed. For each value of *N*_ave_, four covariance matrices were constructed: **C**_act_ was created using data recorded during the ON period of source 1 (i.e. the first 0.5 s of each trial). **C**_pass_ was created using data recorded during the OFF period of source 1 (i.e. 0.5 s < *t* < 1 s). A mean covariance, **C**_*m*_, of the whole trial was also calculated as previously, and this contained information on both source 1 and source 2. A regularised version of **C**_*m*_ was also constructed using the regularisation strategy summarised by Eq.(11). Beamformer weighting parameters were derived using the mean regularised covariance and a lead field tuned to the location of source 1. This meant that for the purposes of this simulation, source 1 could be thought of as the source of interest whereas source two was thought of as interference. For each value of *N*_ave_ the beamformer estimated source power, and source timecourse were computed at the location of source 1. Source power estimates for the ON and OFF periods were plotted as a function of *N*_ave_. To test the accuracy of the timecourse estimate, the correlation between the beamformer estimated timecourse and the original simulated timecourses of both sources 1 and 2 was calculated and plotted as a function of *N*_ave_. Finally, the spatial distribution of the pseudo-T-statistic was plotted.

All of the above was done for both Gaussian and experimental noise and for all computations the accuracy of the estimated *δ* was noted.

**Fig. 2:**
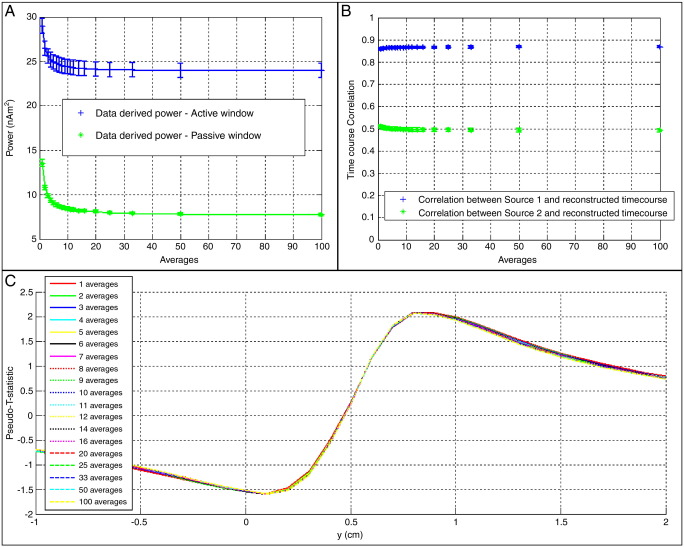
Results of the two source simulations with Gaussian random noise. (A) beamformer reconstructed power in the ON (blue) and OFF (green) periods reconstructed at the location of source 1. Results are plotted against *N*_*ave*_ (B) timecourse correlation. The blue data points show the Pearson correlation coefficient between the beamformer timecourse estimate made at the location of source 1, and the original simulated timecourse for source 1. The green data points show the Pearson correlation coefficient between the beamformer timecourse estimate made at the location of source 1, and the original simulated timecourse for source 2. (C) Example 1-dimensional images, from a single iteration of the simulation, showing the pseudo-T-statistic as a function of position, *y*. Source 1 is at *y* = 1. Source 2 is at *y* = 0. Note for A, B and C, the amplitudes of sources 1 and 2 were 5 nAm and 10 nAm respectively.

Fig. 2 shows results of the two source simulation with Gaussian noise. Fig. 2A shows the beamformer reconstructed power in the ON (blue) and OFF (green) periods reconstructed at the location of source 1. Results are plotted against *N*_ave_. If the beamformer were completely suppressing source 2, then the green line should remain at zero, and the blue line should remain at 25 nAm^2^. The plot therefore shows some improvement with source 2 being better suppressed as the total number of averages is increased. This result is echoed in the timecourse correlation shown in Fig. 2B. Here, the blue data points show the Pearson correlation coefficient between the beamformer timecourse estimate made at the location of source 1, and the original simulated timecourse for source 1. The green data points show the Pearson correlation coefficient between the beamformer timecourse estimate made at the location of source 1, and the original simulated timecourse for source 2. Ideally the blue line should tend towards 1 whereas the green line should tend towards zero. Again a modest improvement is observed with data averaging. Fig. 1C shows a one-dimensional beamformer image. Note that the image is constructed using Eq. (12) where *P*_ON_ is calculated for the first 0.5 s of each trial and *P*_OFF_ is calculated for the last 0.5 s of each trial. Since source 2 is active during the final 0.5 s of each trial, the pseudo-T-stat at the location of source 2 (*y* = 0) is negative.

**Fig. 3:**
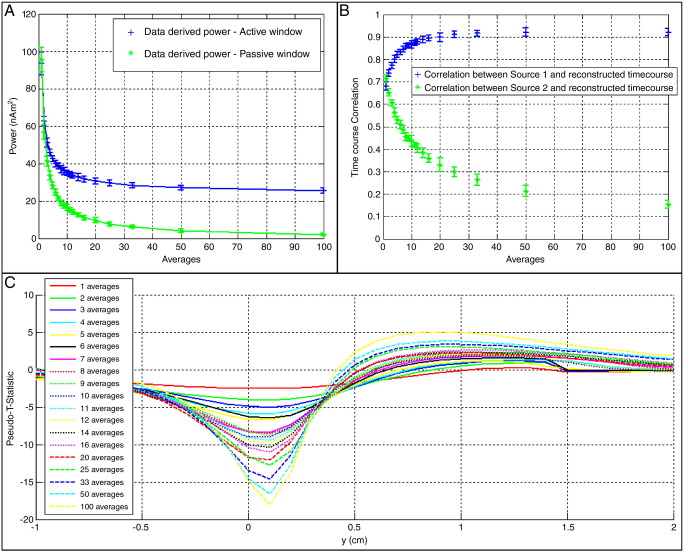
Results of the two source simulation with *Q*_1_ = 5 nAm, *Q*_2_ = 10 nAm, and experimentally measured noise. (A) beamformer reconstructed power in the ON (blue) and OFF (green) periods reconstructed at the location of source 1. Results are plotted against *N*_*ave*_ (B) timecourse correlation. The blue data points show the Pearson correlation coefficient between the beamformer timecourse estimate made at the location of source 1, and the original simulated timecourse for source 1. The green data points show the Pearson correlation coefficient between the beamformer timecourse estimate made at the location of source 1, and the original simulated timecourse for source 2. (C) Example 1-dimensional images, from a single iteration of the simulation, showing the pseudo-T-statistic as a function of position, *y*. Source 1 is at *y* = 1. Source 2 is at *y* = 0.

Fig. 3 shows results of the two source simulation with experimentally recorded noise. A striking difference is observed in terms of the effect of data averaging in the Gaussian noise case, and the experimental noise case. Fig. 3A shows the power in the ON (blue) and OFF (green) periods reconstructed at the true location of source 1. Notice that with no averaging, the power leaking into source 1 is so great that the power estimates in the ON and OFF periods cannot be distinguished. As the level of averaging is increased, the beamformer weights become more spatially specific and are better able to cancel out interference. The timecourse correlation plots shown in Fig. 3B support this argument. Using unaveraged data results in a timecourse estimate with equivalent correlation to both source 1 and source 2. In other words, both sources are indistinguishable. Conversely, with averaged data the timecourse better estimates that of source 1 and correlation with source 2 is low. Fig. 3C shows one-dimensional images equivalent to those in Fig. 2C. Again the advantage of data averaging becomes apparent. The pseudo-T-statistic is based on power difference between the ON and OFF periods, since using unaveraged data the power estimates in these two temporal windows are indistinguishable, no peak occurs in the pseudo-T-statistical image.

As stated above the scalar beamformer involves a search for the optimum azimuthal angle, *δ*, in the tangential plane. Here, using Gaussian noise, for all values of *N*_ave_, this angle search produced a correct result (*δ* = 25 ± 1°). When using real brain noise, the higher signal to noise afforded by data averaging yielded slightly better estimation of *δ* for averaged (*δ* = 25 ± 5°) compared to unaveraged (*δ* = 29 ± 6°) data. This effect was however not thought be a direct cause of the differences between noise models.

The difference between the Gaussian and experimentally recorded noise models can be understood if one considers the dominant noise sources in both cases, and how the beamformer distributes spatial degrees of freedom. In the case of experimentally recorded brain noise, a large number of resting state neural generators will be active leading to a large number of brain sources distributed widely across the cortex. These sources create spatially correlated signals at the channel level and must be rejected by the beamformer. Conversely, Gaussian noise is uncorrelated across channels and does not emulate brain sources. None of the resting state neural mechanisms, measured in experimental data, will be time locked to the trial onset. Averaging will therefore suppress such effects, and the beamformer must try to reject interference from fewer sources in averaged compared to unaveraged data. Previous work (Barnes and Hillebrand, 2003) has shown that the beamformer optimally distributes spatial degrees of freedom such that variations in weighting parameters are greatest close to areas of maximal activation. In other words, the greatest spatial resolution is available close to brain sources. Using averaged data the beamformer can concentrate more spatial degrees of freedom on the suppression of source 2 than it can in unaveraged experimental data. This redistribution of the degrees of freedom acts to increase the spatial specificity of the weights, leading to a more accurate estimate of source dynamics. In the Gaussian noise model, only two sources were simulated. Both were active in averaged and unaveraged data so we assume the degrees of freedom were distributed equally in both cases.

## Retinotopic mapping experiment

Insights from simulation were employed in an experiment designed to investigate spatial specificity of MEG and the beamformer formulation. To do this, the well known retinotopic organisation of the visual cortex was exploited. As an object moves in the visual field, cortical cells at different locations in the visual cortex are activated sequentially, giving the impression of a moving electrical source. The dynamic nature of such a source makes source localisation challenging. In addition the extraction of a timecourse estimate from a single retinotopic location is difficult because signals generated from neighbouring retinotopic sources act as interference. This experiment is therefore similar to our two source simulation in which multiple cortical locations are sequentially activated and the challenge is to reject interference from sources other than that at the location of interest. Four healthy subjects took part in the study, which was approved by the University of Nottingham Medical School ethics committee.

### Experimental method

The visual stimulus (implemented in PsychoPy (Peirce, 2007) www.psychopy.org) comprised a 45° wedge, rotating smoothly around the visual field. The wedge contained a black and white radial checkerboard pattern alternating at a frequency of 10 Hz and extending by a total of 6° from central fixation. (Each element of the checkerboard subtended an azimuthal angle of 15° and extended 2° radially from fixation.) This wedge was presented on a medium grey background. A single trial comprised one full rotation (25 s) followed by 5 s rest and in total 30 trials were presented. MEG data were again recorded using a 275 channel CTF MEG system in third order gradiometer configuration at a sampling rate of 600 Hz. As previously the location of the subject's head was measured by energising and localising 3 fiducial coils inside the scanner. Coil locations were then digitised along with the subject's head shape (*Polhemus isotrack*) and this was fitted to the head surface extracted from 7 T anatomical MR images (parameters as above) in order to gain the locations of the MEG sensors with respect to the brain anatomy.

The 10 Hz flashing checkerboard was expected to drive a 20 Hz phase-locked response in the visual cortex. All MEG data were therefore bandpass frequency filtered between 18 Hz and 22 Hz. They were then processed using beamformer techniques applied using Matlab.

To spatially localise retinotopic locations, Ŧ-statistical images were created by comparison of 18–22 Hz power in active and control time windows. The duration of these time windows was optimised to ensure that enough data was included to obtain a reasonable covariance estimate (see Appendix II). This resulted in a covariance duration *T*_cov_ = 5 s for both the active and control windows. The active time window was allowed to shift in time, and 25 separate pseudo-T-statistical images were constructed using 25 temporally shifted overlapping active windows. These windows were centred at 1 s intervals between 0 and 24 s. For active windows close to the beginning or end of stimulation, the window was allowed to wrap to obtain the required 5s of data. So for example, if a window was centred at *t* = 1 s, data in the range 23.5 s ≤ *t* ≤ 25 s and data in the range 0 s ≤ *t* ≤ 3.5 would be concatenated. For all 25 pseudo-T-statistical images the control time window was taken as the 5 s of rest at the end of each trial, and thus did not overlap with any portion of the active window. In the first case, covariance estimates for the active and control time windows were constructed based on data that was averaged across trials. In the second case, data were left unaveraged and active and control data segments concatenated prior to covariance calculation. In the case of averaged data, resulting covariance matrices were regularised according to the strategy outlined in Eq. (11). The parameter $s_{C_{m}}$ was taken as the minimum singular value of the covariance matrix derived from averaged data in the active and control window. The parameter *s*^unav^ was defined as the minimum singular value of a covariance matrix constructed from the unaveraged, frequency filtered dataset (i.e. using all 30 s of all 30 trials).

Timecourse estimates were made for 5 locations of interest in the visual cortex derived from 5 of the pseudo-T-statistical images described above. These images showed activation in 5 s windows centred at *t* = 2.5 s, 7.5 s, 12.5 s, 17.5 s, and 22.5 s. In all cases two timecourse estimates were made using averaged and unaveraged data. For both, a sliding covariance window technique, similar to that described by Dalal et al. (2008) was employed. The aim of the sliding window technique was to optimise the spatial specificity of the beamformer weights calculation. The covariance window duration, *T*_cov_, was set to its minimum possible value of 5 s. A total of 25 separate covariance windows were used, windows being centred at 1 s intervals between 0.5 s and 24.5 s. The timecourse estimate for the central second within each window was constructed as per Eq. (1).

• For example: the timecourse estimate for the period (5 s ≤ *t* < 6 s) was given by the equation *Q̂*_**θ**_ (5 s ≤ *t* < 6 s) = ***W***(3 s ≤ *t* < 8 s)_**θ**_^T^ ***m***(5 s ≤ *t* < 6 s), where the weights vector ***W***(3 s ≤ *t* < 8 s)_**θ**_ represents the beamformer weights at location and orientation ***θ*** constructed using an average covariance matrix derived from matrices constructed using data in the 3 s ≤ *t* < 8 s window, and data in the control window.

Basing covariance windows on reduced time windows helps to sensitise the weights and ‘focus’ the degrees of freedom on sources active only during this limited period, and not the full 30 s trial length. Reducing *T*_cov_ further would, in principle further improve spatial specificity. However, as shown in Appendix II this may reduce accuracy. Following computation, all covariance matrices were regularised according to Eq. (11). Final timecourse estimates were Hilbert transformed, and the absolute value of the analytic signal computed in order to yield the envelope of oscillatory activity in the 18–22 Hz frequency band.

In order to assess the robustness of temporal effects across the group, timecourses from peak locations in individual subject functional images were derived from beamformer weights constructed using averaged and unaveraged data. These timecourses were median filtered (filter width 0.33 s), averaged across subjects and plotted with error bars showing standard deviation across subjects. Structural images were spatially transformed to a template brain using FLIRT in FSL (http://www.fmrib.ox.ac.uk/fsl/flirt/index.html). This transform was applied to all functional images and the mean location of the peaks in pseudo-*T*-statistical images across the group was derived and marked on the template. It is well known that large inter-individual differences are observed in retinotopic maps and averaging maps across subjects may be misleading. These peak locations therefore only show a crude estimate of approximate brain locations from which timecourses are derived.

In order to quantify the improvement in spatial specificity afforded by data averaging, a simple model of the expected sequential activation of the 5 retinotopic locations was constructed. The model comprised 5 timecourses, and in all cases activation was modelled as a 6.5 s long positive going lobe of a sinusoidal function with a period of 13 s. This function was centred on *t* = 2.5 s, 7.5 s, 12.5 s, 17.5 s, and 22.5 s resulting in 5 timecourses with sequential modulation. A Pearson correlation coefficient between the experimental timecourses (from individual subjects) and a model was computed, along with standard error across subjects.

## Results

**Fig. 4:**
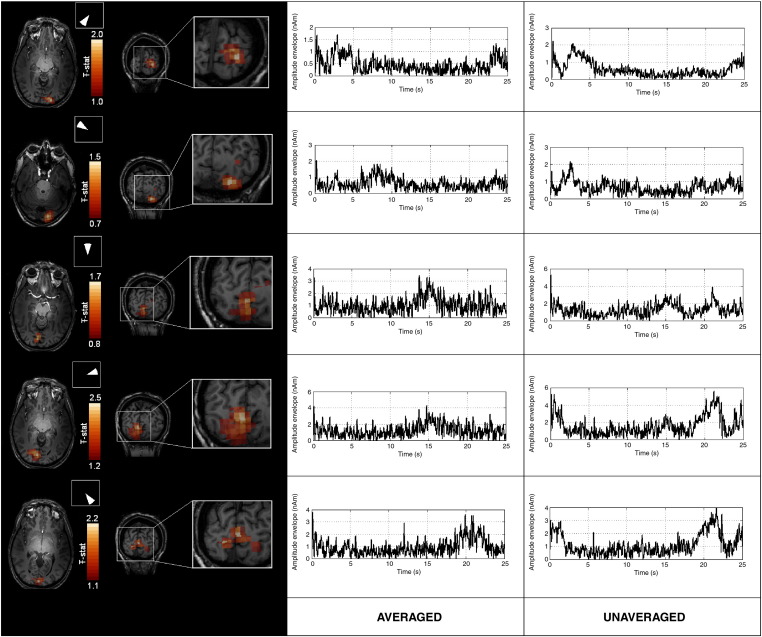
Results of the retinotopic mapping experiment taken from a single subject and derived using averaged data. The left hand column shows pseudo-T-statistical images (derived from averaged data) depicting the retinotopic location of the source. Covariance windows were centred on 2.5 s, 7.5 s, 12.5 s, 17.5 s, and 22.5 s. The location of the stimulus in the visual field at these times is shown inset. The centre and right hand columns show the timecourse estimates of the envelope of 18–22 Hz power. These estimates were taken from peak locations in the images. Timecourses in the centre column are reconstructed using beamformer weights based on averaged data. Timecourses in the right hand column are reconstructed using beamformer weights based on unaveraged data.

Fig. 4 shows the results of the retinotopic mapping experiment for a single representative subject. The left hand column shows pseudo-*T*-statistical beamformer images depicting the retinotopic location of the source. The images shown were based on covariance estimated using averaged data and active windows were centred on 2.5 s, 7.5 s, 12.5 s, 17.5 s, and 22.5 s. The location of the stimulus in the visual field at these times is shown inset. The source location behaves as expected: it switches hemisphere as the stimulus moves from left to right in the visual field. It also moves below the calcarine fissure when the stimulus is in the upper half of the visual field, and above the calcarine fissure when the stimulus is in the lower visual field. This is consistent with the known organisation of the visual cortex and has previously been shown using fMRI. Interestingly, the peaks in pseudo-*T*-statistical images did not change by more than twice the voxel dimension when covariance was constructed using unaveraged data, indicating that averaging has little effect on localisation.

The centre and right hand columns of Fig. 4 show the time evolution of the envelope of 18–22 Hz power. These temporal estimates were reconstructed for locations at the peaks in the functional images. Timecourses in the centre column are reconstructed from beamformer weights based on averaged data. These show clearly the sequential activation of the different retinotopic locations within the cortex. The timecourse from each separate location shows an increase and decrease in oscillatory activity, which occurs as the moving retinotopic source passes that particular location.

It should be pointed out that the temporal scale of the rise and fall of activity in these timecourse estimates is not reflective of the temporal resolution of MEG, but rather the spatial resolution of MEG. As the retinotopic source approaches the location of interest, some power will begin to leak into the source estimate. The temporal scale of the increase in power is therefore dependent on the spatial scale of the power leakage, hence the spatial specificity of the weights, and not the temporal resolution of MEG. Timecourses shown in the right hand column of Fig. 4 are reconstructed using unaveraged data and do not show the same temporal signature as those reconstructed using weights based on averaged data. They appear to be dominated by power changes at the beginning and the end of the stimulation period. Interestingly, the location of the wedge at the beginning and the end of the stimulation period is known to evoke activity in the upper, shallower regions of the visual cortex (i.e. those regions above the calcarine fissure). The implication is that the beamformer weights, computed using unaveraged data, lack the spatial specificity required to cancel out signals from these shallow sources which will have larger SNR simply due to geometry. Without averaging the beamformer cannot therefore accurately reconstruct the temporal signature of these deeper sources.

**Fig. 5:**
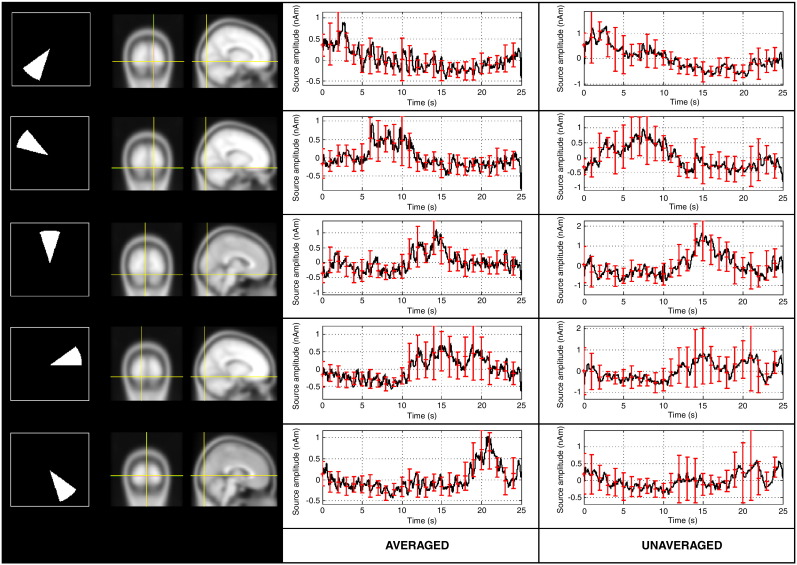
Results averaged across subjects. The left hand column shows averaged locations for each stimulus location. The centre and right hand columns show timecourse estimates reconstructed using beamformer weights based on averaged and unaveraged data respectively. These timecourses are averaged across subjects and error bars show standard deviation.

**Fig. 6:**
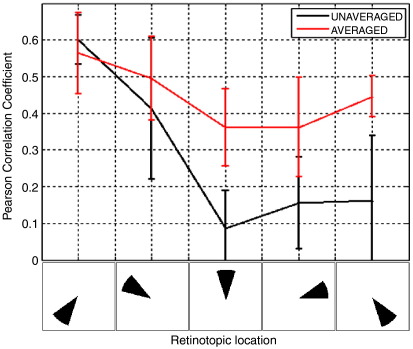
Pearson correlation between the data derived timecourses and the sequential activation model.

Fig. 5 shows the results of the retinotopic mapping experiment, averaged across subjects. Both the spatial and temporal results agree with the single subject result, showing the robustness of the effects that are described. The spatial maps demonstrated the retinotopic organisation of the visual cortex in all subjects. Further, the sequential activation of 5 locations of interest could be observed when timecourse estimates were constructed using weights based on averaged data. This sequential signature was lost when weights were constructed using unaveraged data. This is confirmed in Fig. 6 which shows the Pearson correlation between the experimentally derived timecourses and the sequential activation model for all 5 retinotopic locations. As shown, better correlation was observed for beamformer projected averaged data compared to beamformer projected unaveraged data at four out of the 5 retinotopic locations. The difference in correlation coefficients between averaged and unaveraged data for the 5 retinotopic locations were − 3(± 13)%, 8(± 22)%, 28(± 15)%, 20(± 18)%, and 28(± 18)%.

## Discussion

The initial aim of this study was to address the question: does data averaging improve the spatial specificity of MEG beamformer imaging. Averaging MEG data across trials obviously improves the signal to noise ratio of the measured effects, thus improving the accuracy of covariance estimates used. However, averaging also reduces the total amount of data (information) that is available to construct the covariance estimate which has been shown to degrade accuracy (Brookes et al., 2008). Furthermore the reduction in white noise that is afforded by data averaging can cause data covariance matrices to become ill-conditioned such that the inverse covariance matrix is inaccurate. The problems brought about by data averaging have led previously described methodologies to rely on unaveraged broadband weights (Robinson, 2004; Cheyne et al., 2006).

Our analytical and single source simulation results have shown that, within the limitations of a single source and uncorrelated Gaussian sensor noise model, averaging data has little effect on the final accuracy of beamformer estimates. We showed that, overall, the accuracy of the covariance matrix was improved by data averaging. However, without regularisation, the loss of rank due to averaging caused the inverse covariance matrix to become inaccurate, and therefore beamformer source estimates were distorted severely. To correct for this, a matrix regularisation strategy was introduced. Following regularisation, the noise that was eliminated by averaging was effectively added by regularisation, and therefore there was little or no advantage to averaging across trials. These single source simulation results were echoed by a two source simulation that also employed sensor level Gaussian random noise.

A marked difference was found when experimentally measured broadband ‘brain noise’ was employed. In the Gaussian noise model, the dominant source of noise is obviously uncorrelated across MEG sensors. However, it is well known that in real MEG experiments, evoked stimulus-induced signals are supplemented by interference from both external devices and sources of no interest in the brain. This correlated interference from other sources must be eliminated by the beamformer if a source of interest is to be reconstructed accurately. Averaging across trials acts to reduce (or eliminate) sources of interference that are not phase-locked to stimulus onset. This gives the beamformer the opportunity to ‘focus’ its power on the remaining phase-locked interference, which is then eliminated more effectively. Our two source simulation with experimental brain noise showed that, using beamformer weights based on unaveraged data, the two sources could not be separated. However, they were separated using beamformer weights based on averaged data. This result was echoed in a retinotopic mapping MEG experiment. We tracked the retinotopic movement of a moving electrical source. Further, we showed that, using beamformer weights based on averaged data, independent timecourse estimates could be extracted from locations in the visual cortex in close proximity. These timecourses showed the sequential activation of neighbouring regions in the visual cortex and this sequential signature was not observed when the beamformer weights were computed using unaveraged data.

The ultimate limit to the spatial specificity of MEG is characterised by the ability to extract two independent temporal signals from two cortical locations with a small separation. Using a beamformer technique this is characterised by the spatial specificity of the weights and it is surprising that marked changes in spatial specificity can be observed by making small changes to data covariance estimation. We have shown that spatial specificity is improved by using data averaging to reduce the total number of sources that are to be suppressed by the beamformer. Other ways of changing spatial specificity include reducing the duration of the covariance window, and reducing bandwidth (Dalal et al., 2008). Reducing the duration allows the beamformer to focus on sources active during a limited time window. Frequency filtering to reduce bandwidth allows focusing only on sources within a given frequency band. Many previous studies have used frequency filtered, temporally windowed data to obtain functional images, and then broadband data from the whole experiment to compute a timecourse. Whilst this is not incorrect, it should be pointed out that the weights used for computation of the statistical image and timecourse will have very different spatial specificities. In general, small time frequency windows will allow more focused weights; however, previous work (Brookes et al., 2008) has shown that the accuracy of covariance estimates is degraded if windows are made too small. A trade off is therefore introduced and judicious selection of a strategy to compute data covariance is imperative if the effectiveness of the beamformer estimate is to be optimal. In Appendix II of this paper, a very simple strategy is outlined to achieve accurate beamformer estimates, and still ensure enough information for covariance construction.

Here, averaging MEG data across trials has been shown to improve spatial specificity. There are however limitations to this approach which may mean that it is not always desirable to average. Gaining an accurate source estimate using averaged data is dependent on regularisation. Here we present an approach based on equalising the range of singular values across the two datasets. This strategy adds noise, however averaging and regularising in this way can be thought of as removing correlated interference, and adding uncorrelated Gaussian noise. The latter does not require cancellation by the beamformer. Unfortunately, as the spatial specificity of the estimate improves, the beamformer becomes less robust to inaccurate forward solutions. This means that non-dipolar sources (for example an extended source) may be suppressed if data is averaged prior to covariance computation. It is also conceivable that eliminating the non-phase-locked components of some sources may lead to two spatially separate sources becoming more temporally correlated. This would also lead to suppression of these sources by the beamformer (Sekihara et al., 2002a), and another approach must be employed for accurate localisation. It is certain that in some cases it is not desirable to average data prior to beamformer application and we *do not* recommend that this approach be adopted as a standard. However, in cases where sources are expected in close proximity, and high spatial resolution is required, we can recommend the use of the averaging approach.

Our retinotopic mapping paradigm elicits a phase-locked driven response in the visual cortex. This driven response is narrow-band and relatively long (5 s) time windows were employed to derive pseudo-*T*-statistical images. This paradigm was effectively used to demonstrate the improvement in spatial specificity afforded by data averaging. However, the response localised here is not necessarily representative of the more common broadband brief transient evoked responses often studied in MEG. For this reason further investigation is required to realise the benefits of averaging transient broadband evoked responses for beamformer localisation. The retinotopic mapping paradigm does however represent an ideal way to test the spatial specificity of weighting parameters. This is because sources in close proximity are sequentially active, and to obtain an accurate temporal profile at any one retinotopic location, the source localisation methodology must reject signals from neighbouring sources. Here we show that beamforming affords sufficient spatial specificity to extract the correct temporal sequence of activity from neighbouring retinotopic locations. In addition, this paradigm allows insights into how beamformers might be used to track moving dipolar sources. We have shown that there is a limit to the temporal resolution of beamformer source localisation. This is brought about due to the fact that the covariance window duration, *T*_cov_ must be made large enough to ensure accurate power reconstruction. We outline a simple technique to measure the limits of temporal resolution in Appendix II. We suggest that this approach be used when measuring moving sources, or when using a time frequency beamformer approach. Finally, we suggest that this paradigm be used to test and compare new source localisation techniques. The fact that MEG can achieve the spatial resolution required to track our moving retinotopic source is impressive. Future work should investigate the spatial specificity of MEG using similar paradigms in somatosensory, motor and auditory cortices. The latter is of particular relevance. The quiet environment of the MEG scanner is ideal to investigate tonotopic mapping, something that is difficult in fMRI due to acoustic noise.

## Conclusion

This study has demonstrated that the spatial specificity of MEG beamformer estimates of electrical activity are affected by the way in which covariance estimates are calculated. Previous analytical and simulated results have shown that beamformer estimates are affected by narrowing the time frequency window in which covariance estimates are made (Brookes et al., 2007; Dalal et al., 2008). Here we build on this work by investigating the effect of data averaging prior to covariance estimation. In appropriate circumstances, averaging was shown to lead to a marked improvement in spatial specificity. However the averaging process results in ill-conditioned covariance matrices, thus necessitating an appropriate regularisation strategy. Further, averaging may worsen the problems associated with beamformers and correlated sources, and make beamformers less robust to extended (non-dipolar) sources. We therefore do not recommend averaging as a general rule. However, we have shown that when spatially separate brain sources are active in close proximity, averaging significantly improves the beamformers ability to extract temporally independent signals from locations of interest. A moving visual stimulus was used to elicit brain activation at different retinotopic locations in the visual cortex (Engel et al., 1994). This gives the impression of a moving dipolar source in the brain. We showed that the moving source can be tracked in the cortex. Timecourse estimates were extracted from neighbouring locations of interest in the visual cortex. The sequential activation of separate retinotopic locations was observed with averaging but not without averaging. Finally, the retinotopic paradigm represents an ideal platform to test the spatial specificity of MEG localisation strategies. We suggest future comparisons of MEG localisation techniques could be made using this approach.

## Figures and Tables

**Fig. A1:**
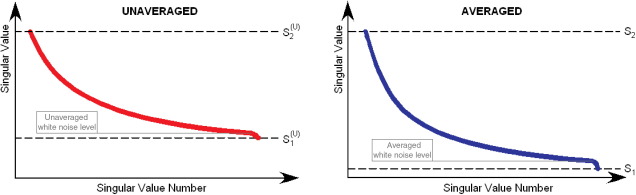
Schematic diagram showing the change in the range of singular values caused by data averaging prior to covariance matrix computation. Note that in both cases, the least significant singular value will underestimate white noise level, however, this underestimation will be far more pronounced in the averaged case.

**Fig. A2:**
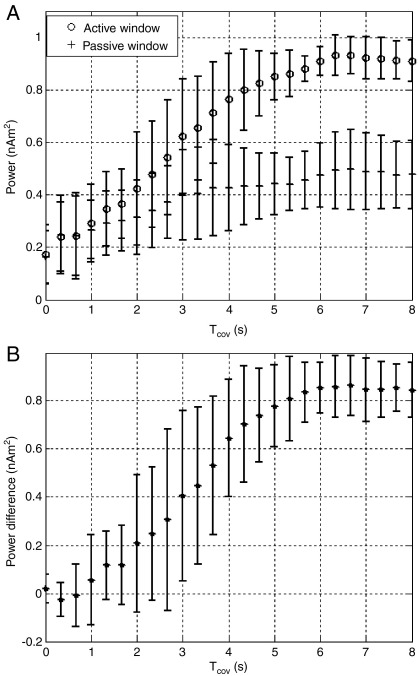
(A) Reconstructed power in the active and control covariance windows versus covariance window duration. (B) Difference in power between active and control windows. Results are averaged across all subjects and two locations.

## Appendix I—Regularisation

The reduction in random noise afforded by averaging can cause the condition number of covariance matrices to increase. In some cases covariance matrices can become ill-conditioned, or singular. It is therefore desirable to regularise the covariance matrix prior to matrix inversion. In this appendix we introduce a strategy for regularisation that ensures a well conditioned matrix.

Fig. A1 shows schematically the spread of singular values for the averaged and unaveraged case. Let **C**_*m*_ represent the covariance matrix constructed from averaged data whilst **C**^(unav)^ represents the covariance matrix based on unaveraged data.

Further, we let the minimum singular value of **C**_*m*_ be represented by *s*_1_ and the maximum singular value of **C**_*m*_ be represented by *s*_2_. Similarly the minimum and maximum singular values of **C**^(unav)^ are denoted by *s*_*1*_^(*u*)^ and *s*_*2*_^(*u*)^ respectively. If we assume that the maximum singular vector of both covariance matrices represents the same phase-locked effect of interest, then *s*_2_ and *s*_2_^(*u*)^ will be equal. In the infinite integration limit, the least significant singular value in both the unaveraged (*s*_1_^(*u*)^) and averaged (*s*_1_) cases would be representative of the white noise level before and after data averaging respectively and the two would be related such that $s_{1}=s_{1}^{\left(u\right)}/\sqrt{N_{\text{ave}}}$. In the case of a finite number of samples however, these least significant singular values will drop below the white noise level. In the case of *s*_1_^(*u*)^, this effect will be slight since data are unaveraged and the number of samples is large. This means that *s*_1_^(*u*)^ remains a useful estimate of white noise level. However, in the case of *s*_1_, the number of samples used is less and so the effect will worsen meaning that *s*_1_ significantly underestimates white noise and $s_{1}<<\ll s_{1}^{\left(u\right)}/\sqrt{N_{\text{ave}}}$. For extremely small number of samples in the averaged data it is possible that *s*_1_ → 0.

The regularisation strategy that we describe involves making the singular value range of regularised **C**_*m*_ equal to the singular value range of **C**^(unav)^. This strategy ensures that the final **C**_*m*_ is well conditioned. Note that we will describe a general case which also allows for regularisation of **C**^(unav)^.

Let the regularised **C**^(unav)^ be given by:

(A1) $$C_{\text{reg}}^{\left(\text{unav}\right)}=C^{\left(\text{unav}\right)}+\mu v^{2}I$$

The matrix condition number is defined as the maximum singular value, divided by the minimum singular value and represents a measure of the range of singular values. Using Eq. A1 and the above notation, the condition number of the regularised matrix constructed using unaveraged data is:

(A2) $$CN_{\text{reg}}^{u}=\frac{s_{2}^{u}+\mu v^{2}}{s_{1}^{u}+\mu v^{2}}$$

The regularised matrix constructed from averaged data is given by

(A3) $$C_{m\text{reg}}=C_{m}+rI$$

and its condition number is given by

(A4) $$CN_{\text{reg}}=\frac{s_{2}+r}{s_{1}+r}$$

To match the range of singular values for the regularised **C**_mreg_ and **C**_reg_^(unav)^, we must derive a regularisation value *r* such that *CN*_reg_ =*CN*_reg_^*u*^. Assuming that *r* << *s*_2_ and that *μν*^2^ << *s*_2_^*u*^, which will be the case for data with appropriately high SNR, then we can write that:

(A5) $$r=\frac{s_{2}}{s_{2}^{u}}\left(s_{1}^{u}+\mu v^{2}\right)-s_{1}$$

The regularised **C**_*m*_ is therefore given by:

(A6) $$C_{mreg}=C_{m}+\left(\left(\mu +1\right)s_{1}^{u}-s_{1}\right)I$$

Finally, we can assume that *s*_2_ ≈ *s_2_^u^*. Furthermore, assuming that there are a large number of samples in the unaveraged case we assume that *ν*^*2*^ ≈ *s*_1_^*u*^. The final expression for the regularised covariance matrix then becomes:

(A7) $$C_{m\mathrm{reg}}=C_{m}+\left\{\left(\left(\mu +1\right)s_{1}^{u}-s_{1}\right)\right\}I$$

For all computations in this paper **C**^(unav)^ is well conditioned and *s*_1_^(*u*)^ is deemed to be a suitably accurate estimation of white noise. For this reason the *μ* = 0 and we use a simplified expression **C**_*m*_^(reg)^ = **C***_m_* + (*s*_1_*^u^* − *s*_1_)***I*** to derive the regularised covariance matrix from averaged data. In a case in which *s*_1_^(*u*)^ is not an accurate estimation of white noise, *μ* can be altered accordingly and the validity of this strategy holds.

## Appendix II—Covariance window optimisation

Our previous work (Brookes et al., 2008) has shown that the accuracy of beamformer reconstruction is dependent on how much data is used to construct the covariance estimate. Specifically, if the covariance window duration *T*_cov_, or the bandwidth *BW* are made too small then the electrical power, reconstructed using the beamformer, is underestimated. It is therefore of great importance to ensure judicious selection of *T*_cov_ and *BW*. In the retinotopic mapping experiment, *BW* was set to 10 Hz and the value of *T*_cov_ was optimised to ensure accurate spatial and timecourse reconstruction. Below a simple procedure for this optimisation is outlined.

• An initial guess of *T*_cov_ = 5 s was made. A single pseudo-*T*-statistical image was then constructed contrasting power in active (0 s < *t* < 5 s) and control (25 *s* < *t* < 30 *s*) windows. This image yielded a location of interest in visual cortex.
• *T*_cov_ was varied between 0.32 s and 8 s in 25 steps. For each value of *T*_cov_ the beamformer reconstructed power at the location of interest, was estimated. Power in active (starting at *t* = 0*s*) and control (starting at *t* = 10 *s*) windows was estimated.
• Power in both active and control windows, and the power difference between active and control, were plotted as a function of *T*_cov_.

The above procedure was repeated using an active window starting *t* = 10s and a control window starting at *t* = 0*s*. This second repetition enabled *T*_cov_ optimisation at a different retinotopic location.

Fig. A2 shows the results of the T_cov_ optimisation procedure. Results are averaged across the two retinotopic locations, and all four subjects. A characteristic curve is produced, showing that for small *T*_cov_ power is underestimated, and the power difference between the active and control windows is indistinguishable. As *T*_cov_ is increased, power estimates, and the power difference are increased. These results are in agreement with previous analytical and simulation studies and suggest that the covariance window duration should not be made less than 5 s in order to ensure accurate power reconstruction.
